# Wortmannin, a specific inhibitor of phosphatidylinositol-3-kinase, induces accumulation of DNA double-strand breaks

**DOI:** 10.1093/jrr/rrz102

**Published:** 2020-02-13

**Authors:** Makoto Ihara, Kazuko Shichijo, Satoshi Takeshita, Takashi Kudo

**Affiliations:** 1 Department of Radioisotope Medicine, Atomic Bomb Disease and Hibakusha Medicine Unit, Atomic Bomb Disease Institute, Nagasaki University, Nagasaki, Nagasaki 852-8523, Japan; 2 Department of Tumor and Diagnostic Pathology, Atomic Bomb Disease and Hibakusha Medicine Unit, Atomic Bomb Disease Institute, Nagasaki University, Nagasaki, Nagasaki 852-8523, Japan; 3 Department of Molecular Medicine, Nagasaki University, Graduate School of Biomedical Sciences, Nagasaki, Nagasaki 852-8523, Japan; 4 Joint Research Office, Research Promotion Division, Office for Research Initiative and Development, Nagasaki University, Nagasaki, Nagasaki 852-8521, Japan

**Keywords:** Wortmannin, SCID cells, DNA double-strand breaks, γH2AX, *in vitro* phosphorylation

## Abstract

Wortmannin, a fungal metabolite, is a specific inhibitor of the phosphatidylinositol 3-kinase (PI3K) family, which includes double-stranded DNA dependent protein kinase (DNA-PK) and ataxia telangiectasia mutated kinase (ATM). We investigated the effects of wortmannin on DNA damage in DNA-PK-deficient cells obtained from severe combined immunodeficient mice (SCID cells). Survival of wortmannin-treated cells decreased in a concentration-dependent manner. After treatment with 50 μM wortmannin, survival decreased to 60% of that of untreated cells. We observed that treatment with 20 and 50 μM wortmannin induced DNA damage equivalent to that by 0.37 and 0.69 Gy, respectively, of γ-ray radiation. The accumulation of DNA double-strand breaks (DSBs) in wortmannin-treated SCID cells was assessed using pulsed-field gel electrophoresis. The maximal accumulation was observed 4 h after treatment. Moreover, the presence of DSBs was confirmed by the ability of nuclear extracts from γ-ray-irradiated SCID cells to produce *in vitro* phosphorylation of histone H2AX. These results suggest that wortmannin induces cellular toxicity by accumulation of spontaneous DSBs through inhibition of ATM.

## Introduction

Wortmannin, a metabolite isolated from *Penicillium funiculosum*, is a specific inhibitor of the phosphatidylinositol 3-kinase (PI3K) family [1]. At concentrations of ~20 μM, it can sensitize multiple types of cells to radiation [2]. Boulton *et al.* reported significant correlations between wortmannin concentrations, cell survival and DNA repair after exposure to ionizing radiation [3]. Other studies have shown that wortmannin treatment inhibits growth of tumors [4], inhibits proliferation, induces apoptosis [5] and promotes cell death [6, 7]. Okayasu *et al.* reported that wortmannin reduced plating efficiencies of human cells by up to 30% [8]. We hypothesized that these effects may be caused by DNA damage induced by the wortmannin treatment itself.

DNA double-strand breaks (DSBs) have been shown to be the most critical lethal DNA lesions in cells. They induce tumors if misrepaired, or cell death if left unrepaired. DSBs can be generated during DNA replication, recombination (including V(D)J recombination in the immune system) or by exogenous factors such as ionizing radiation and radiation-mimetic agents, as well as by endogenous factors such as radicals, reactive oxygen species generated by metabolic events, and through the indirect actions of radiation [9, 10, 11]. DSBs can be repaired through two major cellular repair pathways: homologous recombination (HR) and non-homologous end joining (NHEJ) [12, 13]. In mammalian cells, NHEJ is the major repair pathway, in which DNA-dependent protein kinase (DNA-PK) plays an important role [14, 15]. V(D)J recombination is mediated through NHEJ [16].

Severe combined immunodeficient (SCID) mice have a recessive disorder that is characterized by immunodeficiency [17] and defective DNA repair [18]. Therefore, cells isolated from SCID mice are hypersensitive to ionizing radiation relative to cells from wild-type mice [19]. SCID mutation is located at the C-terminus of the gene encoding the catalytic subunit of DNA-PK, DNA-PKcs (c.T12,138A, p.Y4,046X), leading to the loss of 83 amino acid residues at the C-terminus. This mutation greatly destabilizes the DNA-PKcs protein, resulting in undetectable levels of DNA-PKcs expression and DNA-PK kinase activity [20–22].

Ataxia telangiectasia (AT) is a recessive disease characterized by cerebellar ataxia, telangiectasia, immunodeficiency and a predisposition to malignancy [23]. Cells isolated from AT patients exhibit increased radiosensitivity [24]. Ataxia-telangiectasia mutated (*ATM*), the gene responsible for AT, encodes a protein kinase [25]. When DSBs are generated, ataxia telangiectasia mutated kinase (ATM) is activated through autophosphorylation and phosphorylates histone H2AX at serine 139 [26]. Therefore, the number of phospho-histone H2AX (called γH2AX)-positive foci correlates with that of DSBs [27, 28]. γH2AX subsequently recruits repair molecules to the sites of DSBs. Hartley *et al.* described the homology between DNA-PKcs, ATM and PI3K, and were the first to demonstrate that DNA-PK is sensitive to wortmannin [29]. As proteins responsible for DNA damage, including DNA-PKcs and ATM, contain a PI3K motif, they are inhibited by high concentrations of wortmannin [30]. ATM and DNA-PK belong to class IV of the PI3K family [31].

In this study, we investigated the generation of DSBs by wortmannin in cultured cells obtained from DNA-PKcs-deficient, radiation-sensitive SCID mice. Wortmannin inhibits ATM activity, thereby inhibiting the phosphorylation of histone H2AX. Therefore, wortmannin-induced DSBs are not observed in wortmannin-treated cells. To overcome this, we attempted to induce *in vitro* phosphorylation of histone H2AX using nuclear extracts from γ-ray-irradiated SCID cells that lack DNA-PKcs, but have ATM kinase.

## Materials and Methods

### Cells

SCID cells (SC3VA2) [32] and AT cells (AT5BIVA) were cultured in Dulbecco’s modified Eagle’s medium (DMEM; Invitrogen, Carlsbad, CA, USA) supplemented with 10% fetal calf serum (Equitech-Bio, INC. Kerrville, TX, USA).

### Irradiation

Cells were irradiated with a ^137^Cs γ-irradiator (Pony Industry, Chuo-ku, Osaka, Japan) at a dose rate of 1 Gy/min at room temperature. To measure DSBs repair, cells were irradiated with 20 Gy. Wortmannin (20 μM, Sigma-Aldrich, St. Louis, MO, USA) was added to the culture medium 2 h before irradiation.

### Cell survival

Cell survival was measured using a colony formation assay. Briefly, cells in exponential growth phase were treated with 5–50 μM of wortmannin at 37°C for 2 h. Cells were trypsinized and plated onto 100-mm diameter culture dishes. The number of cells plated per dish was optimized to obtain at least 50 colonies. After incubation in the wortmannin-containing medium for 1 day, cells were washed with PBS(−) (137 mM NaCl, 2.7 mM KCl, 10 mM Na_2_HPO_4_ and 1.76 mM KH_2_PO_4_, pH 7.4), and incubated in fresh medium for 2 weeks.

### Measurement of DNA DSBs

Numbers of DSBs were calculated based on the density of bands observed after pulsed-field gel electrophoresis (PFGE). Briefly, cells were treated with 20 μM wortmannin and incubated at 37°C for the indicated periods. Harvested cells were resuspended in PBS at a density of 2 × 10^7^ cells/ml and treated as described previously [33]. An equal volume of 1% agarose was added to the cell suspension. Aliquots (100 μL) were placed in a plug former and solid plugs were incubated with lysis buffer (1 mg/ml protease K and 1% *N*-lauroylsarcosine sodium salt in 0.125 M EDTA, pH 9.0) at 50°C overnight. The resulting plugs were used for electrophoresis.

Plugs were loaded onto 1% SeaKem GTG agarose gels (Cambrex Bio Science Inc., Rockland, ME, USA). Electrophoresis was performed at a field strength of 0.6 V/cm and alternated at 120 s in 0.5× TBE (Tris-borate-EDTA) buffer for 24 h at 9°C in a CHEF-DR II apparatus (Bio-Rad Laboratories Inc. Hercules, CA, USA). Gels were stained for 1.5 h with ethidium bromide (5 μg/ml) and destained for 3 h in 0.5× TBE buffer. Fluorescence intensities were measured using a UV transilluminator from FluorChemR Imaging Systems (Alpha Innotech, San Leandro, CA, USA). The intensity of bands corresponding to fragmented DNA released from the origin was measured.

### Preparation of nuclear extracts

Nuclear extracts were prepared as described by Dignam *et al.* [34] with modifications. Briefly, SCID cells were irradiated with 10 Gy of γ-rays. After incubation at 37°C for 30 min, cells were suspended and disrupted in buffer A (10 mM HEPES-KOH, 10 mM KCl, 0.1 mM EDTA, pH 8.0) with a Dounce homogenizer. Nuclei were separated by centrifugation at 130 × *g* for 5 min. Nuclear extracts were prepared with buffer C (50 mM Hepes-KOH, 420 mM KCl, 0.1 mM EDTA, 5 mM MgCl_2_ and 20% glycerol at pH 8.0). After clarifying by centrifugation (14 000 × *g*, 30 min), the supernatant was stored at −80°C until further use.

### *In vitro* phosphorylation of H2AX

SCID cells (1 × 10^6^) were seeded onto 22 × 22 mm glass cover slips in 60-mm culture dishes. After incubation at 37°C for 2 days, cells were treated with wortmannin (20 μM) for the indicated time periods. Coverslips were then fixed with cold methanol (−20°C) for 10 min, washed with PBS(−), incubated in reaction buffer (20 mM Tris-HCl pH 7.5, 2 mM MgCl_2_, 2 mM ATP, 1 mM DTT and 50 μL nuclear extract) at 37°C for 30 min. After three additional washes with PBS(−), cells were fixed with 4% formaldehyde in PBS(−). For blocking, cells were treated with goat serum at room temperature for 2 h, washed with PBS(−), and incubated overnight with primary rabbit polyclonal anti γH2AX antibody (Sigma-Aldrich, St. Louis, MO, USA) at 4°C, followed by incubation with fluorescein isothiocyanate (FITC)-conjugated anti-rabbit secondary antibody for 1 h at room temperature. Nuclei were counterstained by incubation with 4′, 6-diamidino-2-phenylindole (DAPI). Images were acquired by fluorescence microscopy (Leica Microsystems GmbH, Wetzlar, Germany) and processed with Adobe Photoshop software (Adobe Systems, San Jose, CA, USA). Nuclear γH2AX foci were counted in 100 cells.

## Results and Discussion

### Wortmannin increases radiation sensitivity

**Fig. 1.**
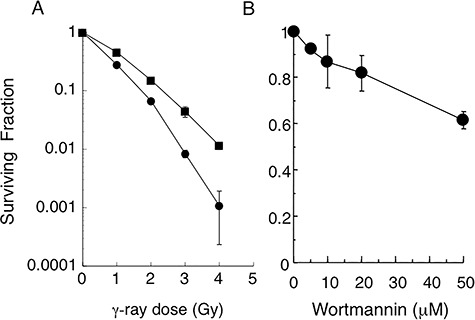
Survival of SCID cells with or without wortmannin treatment. (**A**) SCID cells were untreated (closed squares) or treated with 20 μM wortmannin for 2 h (closed circles) before irradiation. The medium was changed 16 h after irradiation. (**B**) Cells were treated with wortmannin at 0–50 μM for 2 h. After changing the medium, cells were incubated, and colony formation was examined. Data are averages of three independent experiments. Standard errors are indicated at each time point.

We first investigated radiosensitivity of SCID cells with or without wortmannin treatment. As shown in Fig. 1A, the surviving fraction of wortmannin-treated SCID cells was lower than that of the untreated SCID cells. The *D*_10_ (dose at 10% survival) was 2.5 Gy and 2 Gy for untreated and wortmannin-treated cells, respectively. This is likely a result of induction of DSBs or the inhibition of DNA repair systems (possibly HR) other than NHEJ, as SCID cells are deficient in NHEJ. The reduction in survival by wortmannin was consistent with the results of Okayasu *et al.* [8], who reported that wortmannin reduced plating efficiencies by up to 30%.

### Wortmannin decreases cell survival

Figure 1B shows a survival curve for wortmannin-treated SCID cells without irradiation. The surviving cell fraction decreased with increasing concentration of wortmannin (5–50 μM). Based on the survival curves of wortmannin-treated SCID cells (Fig. 1A and B), 20 and 50 μM of wortmannin treatment induced DNA damage equivalent to 0.37 and 0.69 Gy of γ-irradiation, respectively. Survival curves plotted on a semi-log scale were linear. Considering the possibility that wortmannin might induce DSBs, a one-hit equation was used to fit these data, and the lethal dose 50% (LD50) of SCID cells for wortmannin was estimated to be 75 μM. Because it has been reported that wortmannin treatment inhibits growth of cancer [4], inhibits proliferation, induces apoptosis [5] and promotes cell death [6, 7], we hypothesized that wortmannin induces the accumulation of DSBs, leading to cellular toxicity.

### Wortmannin treatment increases DSBs

**Fig. 2.**
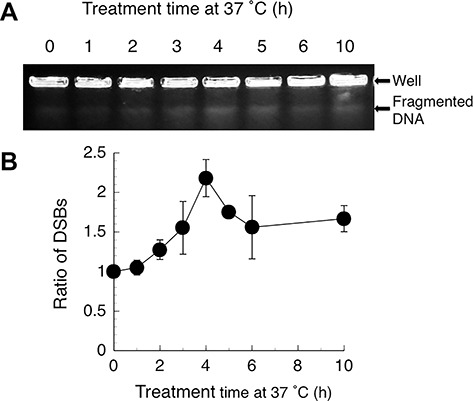
Wortmannin induces DSBs in SCID cells. Cells were cultured with 20 μM wortmannin for the indicated periods. (**A**) DSBs were analysed by PFGE. (**B**) DSBs production over time. Data are averages of three to five independent experiments. The density of DSBs bands observed in non-treated cells was set to a ratio of 1.

DSBs in wortmannin-treated SCID cells were assayed by PFGE. We measured the intensity of bands corresponding to fragmented DNA released from the origin that indicates the amount of DSBs. Because the same number of cells was used in plug preparations, the same amount of DNA was applied to the gel. Hirayama *et al*. reported that the intensity of the DNA band released from the origin increases depending on the radiation dose [35]. We also confirmed that the decrease in intensity correlates with incubation time after irradiation [33]. As shown in Fig. 2A, the number of DSBs increased with time in the presence of 20 μM wortmannin. The maximal relative ratio of DSBs (2.5-fold over control) was observed after a 4 h treatment period, followed by a gradual decrease (Fig. 2B). Rosenzweig *et al.* reported that 20 μM wortmannin inhibits DNA-PK activity in cell extracts, and that this inhibition correlates closely with the observed increase in radiosensitivity [36]. These results are consistent with ours.

### *In vitro* phosphorylation of histone H2AX in wortmannin-treated cells

**Fig. 3.**
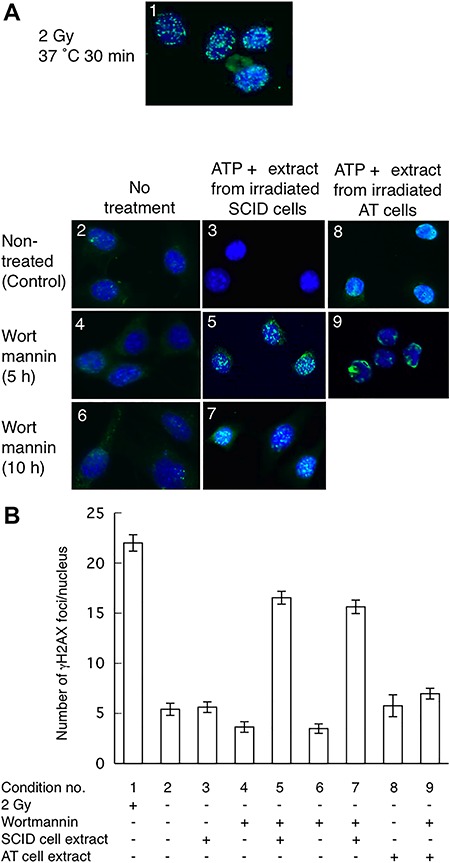
*In vitro* phosphorylation of histone H2AX. (**A**) Typical γH2AX foci observed in the nuclei of SCID cells. (**B**) Numbers of γH2AX foci per nucleus. Panels in (A) and bars in (B) have corresponding numbers designating experimental conditions: 1, SCID cells irradiated and incubated at 37°C for 30 min; 2, no treatment; 3, no treatment with wortmannin and treated with ATP and nuclear extract from irradiated SCID cells; 4, wortmannin-treated (5 h); 5, wortmannin-treated for 5 h and treated with ATP and nuclear extract from irradiated SCID cells; 6, wortmannin-treated (10 h); 7, wortmannin-treated (10 h) and treated with ATP and nuclear extract from irradiated SCID cells; 8, no treatment with wortmannin and treated with ATP and nuclear extract from irradiated AT cells; and 9, wortmannin-treated (5 h) and treated with ATP and nuclear extract from irradiated AT cells. Data are averages of γH2AX foci in 100 cells except in conditions 8 and 9. Conditions 8 and 9 are averages of 25 cells. Error bars indicate the standard error of the number of foci per cell.

The PFGE data suggested that wortmannin treatment induces DSBs accumulation (Fig. 2A and B). γH2AX is a marker for DSBs [28]. When DSBs are generated, either endogenously or exogenously, ATM phosphorylates histone H2AX [26] around DSB ends. Thus, the local activation of ATM and interaction with target proteins are important for nuclear focus formation. Wortmannin inhibits class IV PI3K family members, including ATM and DNA-PKcs [30], thereby inhibiting the phosphorylation of histone H2AX. To overcome this, we attempted to induce *in vitro* phosphorylation of histone H2AX using ATP *(*adenosine triphosphate) and nuclear extracts from γ-ray-irradiated SCID cells. SCID cells lack DNA-PKcs, but have wild-type ATM kinase. Nuclear extracts were prepared from γ-ray-irradiated SCID cells to activate ATM. Nuclear extracts from irradiated SCID cells should contain activated ATM that can further phosphorylate histone H2AX and other ATM substrates. Figure 3A shows representative images of γH2AX staining in SCID cells.

We detected γH2AX by immunofluorescence staining. Fig. 3A, panel 1, and Fig. 3B, condition 1, show representative γH2AX foci formation after 2 Gy of γ-irradiation. γH2AX was not observed in non-wortmannin-treated SCID cells (Fig. 3A, panel 2, and Fig. 3B, condition 2). These results indicate that γH2AX focus formation is specific to irradiation-induced lesions. Moreover, γH2AX was not observed in the non-wortmannin-treated cells treated with ATP and extracts from irradiated SCID cells (Fig. 3A, panel 3 and 3B, condition 3). This is likely because no DSBs exist in non-irradiated cells. This is consistent with H2AX being spread over nuclei but not aggregating around DSBs and with distributed phosphorylated H2AX being unable to form visible foci.

As shown in Fig. 3A, panel 5 and 3B, condition 5 (5 h treatment) and Fig. 3A, panel 7 and 3B, condition 7 (10 h treatment), characteristic γH2AX foci were observed in the wortmannin-treated cells treated with ATP and extracts from irradiated SCID cells. This suggests that ATM kinase in nuclear extracts from the irradiated SCID cells phosphorylates H2AX at DSB ends. However, no γH2AX foci were observed in the wortmannin-treated SCID cells not treated with ATP and extracts from irradiated SCID cells (5 h treatment, Fig. 3A, panel 4 and Fig. 3B, condition 4; 10 h treatment, Fig. 3A panel 6 and Fig. 3B condition 6). This is likely because wortmannin inhibits ATM, and thus, H2AX was not phosphorylated. γH2AX foci were not observed in non-wortmannin-treated cells treated with ATP and nuclear extracts from irradiated AT cells (Fig. 3A, panel 8; Fig. 3B, conditions 8) similar to non-wortmannin-treated cells treated with ATP and nuclear extracts from irradiated SCID cells (Fig. 3A, panel 3; Fig. 3B, conditions 3). On the contrary, clear but small numbers of γH2AX foci were observed in wortmannin-treated cells treated with ATP and extracts from irradiated AT cells (Fig. 3A, panel 9, and Fig. 3B, condition 9). This indicates that irradiated AT cells may have some capacity to phosphorylate histone H2AX. These results indicate that ATM primarily phosphorylates histone H2AX. This time course of appearance of γH2AX foci is consistent with the time course of DSBs levels observed by PFGE (Fig. 2). These results strongly suggest that wortmannin treatment induces the accumulation of DSBs.

In living cells, DSBs are continuously generated during DNA replication [10, 11] and the action of endogenous radicals [37, 38]. HR and NHEJ function to repair these spontaneously induced DSBs. As wortmannin inhibits both DNA-PKcs and ATM, it is hypothesized that spontaneous DSBs accumulate in wortmannin-treated cells. Gu *et al.* reported that wortmannin inhibits the repair of free radical-mediated DSBs in an *in vitro* system using synthetic substrates [39]. Exposure of non-small-cell lung cancer cells to wortmannin inhibited proliferation in a concentration-dependent manner *in vitro* [4]. Extensive DNA fragmentation (laddering) was detected in human prostate carcinoma cells 4–6 h after wortmannin treatment [40]. These reports strongly support the idea that free radical-mediated DSBs accumulate in wortmannin-treated cells.

In the present study, we devised an *in vitro* phosphorylation assay, applying nuclear extract onto fixed cells. This assay might be useful to detect phosphorylated proteins under kinase-inactive conditions as demonstrated here. Moreover, this assay can also visualize the spatial distribution of phosphorylated proteins. The rate of H2AX phosphorylation depends on the batch of nuclear extract. Based on the results of our study, it is likely that survival in wortmannin-treated cells was lower than in untreated cells because wortmannin inhibits both DNA-PKcs and ATM-dependent repair mechanisms, leading to the accumulation of spontaneous DSBs that are not repaired owing to the inhibition of repair mechanisms. The resulting excess DSBs would then increase mutations, owing to the activity of the error-prone DNA repair systems that are not inhibited by wortmannin, potentially giving rise to cancer. Therefore, further studies are necessary to resolve these questions.
